# Impact of the Carbon Nanofillers Addition on Rheology and Absorption Ability of Composite Shear Thickening Fluids

**DOI:** 10.3390/ma13173870

**Published:** 2020-09-02

**Authors:** Paulina Nakonieczna-Dąbrowska, Rafał Wróblewski, Magdalena Płocińska, Marcin Leonowicz

**Affiliations:** Faculty of Materials Science and Engineering, Warsaw University of Technology, Woloska 141, 02-507 Warsaw, Poland; rafal.wroblewski@pw.edu.pl (R.W.); magdalena.plocinska@pw.edu.pl (M.P.); marcin.leonowicz@pw.edu.pl (M.L.)

**Keywords:** shear thickening fluids, nanocomposite fluids, multi-walled carbon nanotubes, carbon fillers

## Abstract

Synthesis and characterization of composite shear thickening fluids (STFs) containing carbon nanofillers are presented. Shear thickening fluids have attracted particular scientific and technological interest due to their unique ability to abruptly increase viscosity in the case of a sudden impact. The fluids have been developed as a potential component of products with high energy absorbing efficiency. This study reports on the rheological behavior, stability, and microstructure of the STFs modified with the following carbon nanofillers: multi-walled carbon nanotubes, reduced graphene oxide, graphene oxide, and carbon black. In the current experiment, the basic STF was made as a suspension of silica particles with a diameter of 500 nm in polypropylene glycol and with a molar mass of 2000 g/mol. The STF was modified with carbon nanofillers in the following proportions: 0.05, 0.15, and 0.25 vol.%. The addition of the carbon nanofillers modified the rheological behavior and impact absorption ability; for the STF containing 0.25 vol.% of carbon nanotubes, an increase of force absorption up to 12% was observed.

## 1. Introduction

A fluid is defined as a substance that flows [1]. All fluids that do not comply with this law are called non-Newtonian, i.e., their viscosity depends on the rate and time of shearing. Some paints or blood are examples of such fluids [2]. Non-Newtonian fluids are divided into the following two groups: Rheologically stable which do not change rheological properties during shearing and rheologically unstable with rheological properties that are a function of shear rate and time.

A shear thickening fluid (STF) is composed of the following two basic components: The continuous viscous component which is often various types of glycols and the rigid component which is often silica powder. A suspension of both components brings about a unique effect of shear thickening, resulting in an abrupt growth of viscosity with increasing shear rate [3,4,5]. Shear thickening fluids display a dilation phenomenon which means that, at a sufficiently high shear rate, their properties change abruptly from that typical of a viscous liquid to the characteristics of an elastic solid [6]. Usually, the STFs exhibit a viscoelastic behavior. The properties of such fluids can be controlled by their composition, which allows them to tailor the final viscosity and critical shear rate at which the initial viscosity abruptly increases [7].

There are several rheological models that, in a more or less precise way, describe the behavior of a particular rheological system in the conditions of low and high shear rates [7]. Each time, an appropriate model is selected to describe a system, it should be accompanied by gathering information on the composition of the fluid and possible interactions among the components of the suspension [8].

**Figure 1:**
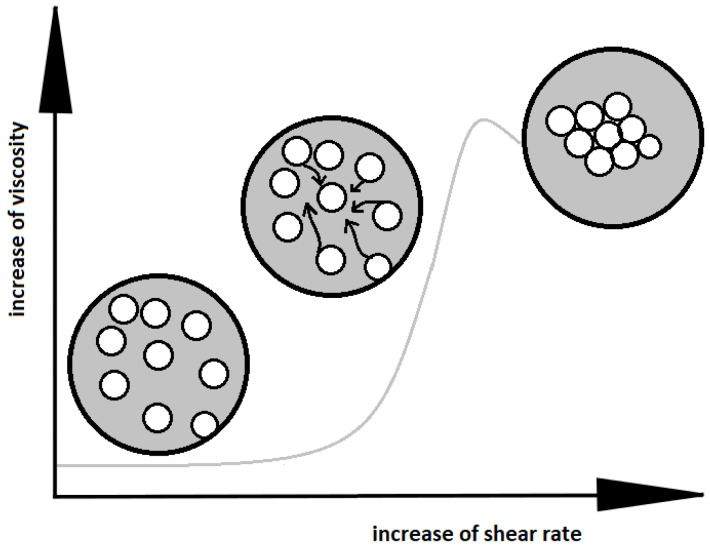
Change of the arrangement of particles in shear thickening fluid with an increase of the shear rate.

The basic mechanisms that describe the phenomena occurring in shear thickening fluids, are primarily based on the frictional forces between the individual solid particles in the suspension [7,9]. According to Reynolds theory, in a basic state, the solid phase particles are softly packed at a low shear rate. The frictional forces between the particles are small, because the liquid between them acts as a lubricant. As the shear rate increases, the distance between the particles decreases (see Figure 1). The slipping ability of the matrix decreases, the friction between the particles increases, as well as the viscosity of the system. Reynolds explained the phenomenon as dilation, based on the change of the volume of the system [7,8]. Another theory that attempts to explain the phenomenon of shear thickening is the order-disorder theory. In agreement with this mechanism, for low shear rates, the solid particles of the STF move in an orderly manner so that the viscosity of the system is low, reflecting the lack of friction between the particles. With an increase of the shear rate, the ordered structure is destroyed, the friction between particles grows, and the viscosity of the system increases. The theory of clusters formation considers mutual hydrodynamic interactions of solid phase particles and collective blocking of their movement as a probable mechanism for the viscosity change [10].

The existing mechanisms facilitate, more or less, an understanding of the relationship between the reinforcement (solid phase particles) and the matrix (liquid phase). It is important to tailor the properties of the STF for a particular application. Shear thickening fluids in combination with high-performance woven fabrics, such as para-aramid fabrics (Kevlar and Twaron) or ultra-high molecular weight polyethylene (Dyneema) have been used for a variety of applications [11,12,13]. The widest application area is related to personal protective products such as helmets, protective sport garments, and smart body armor [14,15,16].

A variety of modifications have been introduced for the fabrication of composite STFs with different rheological properties. Ge et al. modified STFs with SiC nanowires [17]. Other studies have reported a minor addition of graphene and silica microspheres [18,19,20], in which the level of maximal viscosities obtained was below 1000 Pa·s. Carbon additives seem to be the best fillers that has improved the maximal viscosity of STFs and also their stability [21,22,23,24]. For example, it has been reported that the addition of expanded graphite increased the dilatant effect, up to three times, due to the improved packing of the solid phase [25]. 

The current study reports on the rheological behavior, stability, and microstructure of the STFs modified with selected carbon nanofillers, such as multi-walled carbon nanotubes, reduced graphene oxide, graphene oxide, and carbon black. The correlations among the properties and structure of the STFs are presented and discussed.

## 2. Materials and Methods 

For the synthesis of shear thickening fluids, an amorphous silica KE-P50, having spherical particles with diameters ranging from 500 to 600 nm, from Nippon Shokubai (Osaka, Japan), were mixed, in appropriate proportions, with polypropylene glycol PPG2000 from Acros Organics (Geel, Belgium), having a molar mass 2000 g/mol and density of 1 ± 0.01 g/cm^3^. The content of silica in the basic fluid was 55 vol.% This proportion was chosen on the basis of our previous research, in which silica content ranging from 30 to 60 vol.% were tested (see [26]). A 55 vol.% content of silica obtained the maximal peak of the viscosity and allowed the components to effectively disperse.

The composition of STFs was modified by the addition of the following carbon nanofillers: multi-walled carbon nanotubes (MWCNT, Nanocyl NC7000, Sambreville, Belgium), carbon black (CB, The Cary Company, Addison, IL, USA), graphene oxide (GO, Institute of Electronic Materials Technology, Warsaw, Poland), and reduced graphene oxide (rGO, Institute of Electronic Materials Technology, Warsaw, Poland). The properties of the carbon materials used are shown in Table 1. The STFs were modified by minor additions of the nanofillers with the amounts of 0.05, 0.15 and 0.25 vol%. It was impossible to obtain a homogeneous suspension with the higher content of the nanofillers. In order to disperse the particles in the liquid, the calender EXAKT 80E (Oklahoma City, OK, USA) was used. The synthesis of composite shear thickening fluids consisted of the following two major steps:

(1) The mixture of glycol and the carbon nanofiller (in appropriate proportion) was passed through a calender to form a homogenous suspension (without agglomerates);

(2) The mixture of glycol and the carbon nanofiller was added to amorphous silica and mixed in appropriate proportion (know-how protected).

The shape of the carbon nanofillers was characterized using an HITACHI S3500 scanning electron microscope (SEM) (Krefeld, Germany) in SE mode.

The rheological properties were examined using an ARES rheometer (TA Instruments) (New Castle, DE, USA), equipped with two parallel plates (φ 25 mm) with a 0.3 mm gap between them. The size of the gap was optimized for all the fluids, based on previous research. All the viscosity measurements were performed at room temperature.

The STFs with the best rheological properties (highest maximal viscosity) were chosen for the impact test to study the force absorbing efficiency. The synthesized STFs were used for impregnation of the three-dimensional (3D) fabric (polyester three-dimensional woven fabric M8180 from Baltex Ltd. Warsaw, Poland), which was sealed between thin silicon membranes (130 × 30 × 15 mm). The impact tests were carried out using a drop tower, by dropping an impactor, with energy of 5 J, onto the sample (the procedure based on British Standard BS 7971–4:2002). The dependence between the force and time was registered by a force sensor.

**Figure 2:**
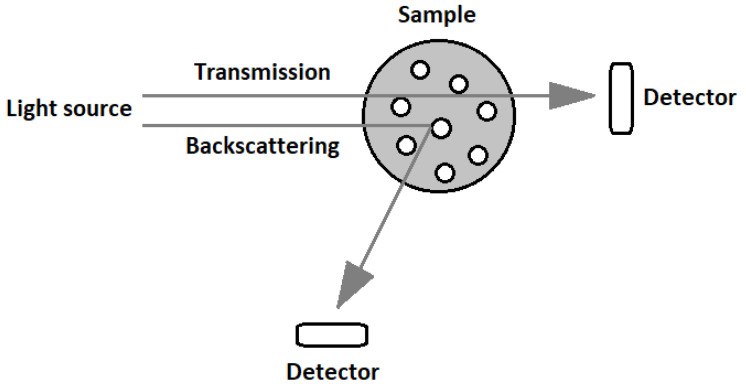
The principle of operation of the Turbiscan device.

The structural stability of the STFs was studied using a Turbiscan Lab analyzer (Formulation, Toulouse, France) with the light λ = 880 nm (see [27]). Some of the STFs samples were tested in a sealed cylindrical glass vial to show the changes in the arrangement of particles over time. The measurements were performed for 370 days at room temperature. The light intensity profiles were achieved with a scan step of 40 µm, along with the entire height of the measuring cell. The Turbiscan Lab has two synchronous detectors for the analysis of liquids (Figure 2). These devices detect the intensity of transmitted and backscattered light over the entire sample. Only part of the light passes through the sample, which is registered by sensors located on the walls of the chamber. The measurement was carried out without any mechanical or external stress, and therefore the true aging of the product was monitored. The resulting profile was plotted as the backscattered intensity of the light passing through the fluids versus the height of the dispersion. All the measurements were taken at room temperature. The peaks from the bottom of the vial and from the top of the unfilled vial were removed from the graph.

## 3. Results and Discussion

### 3.1. Microstructure

**Figure 3:**
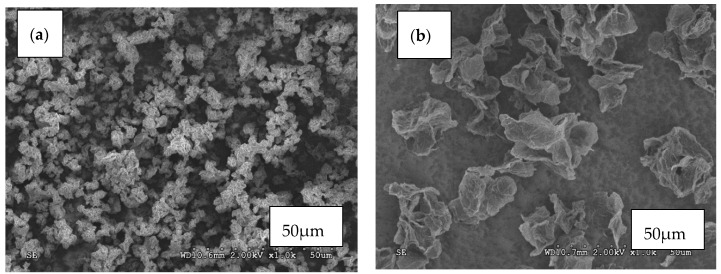

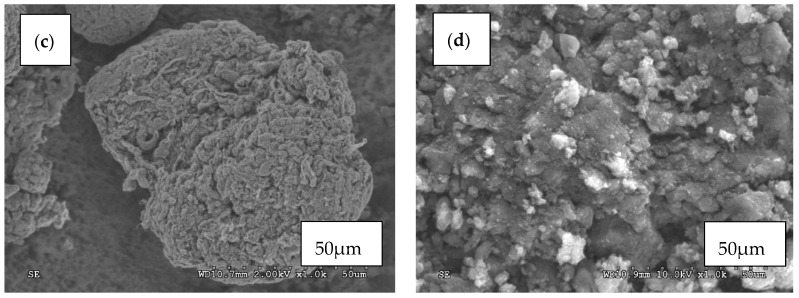
SEM images of (**a**) Graphene oxide GO; (**b**) Reduced graphene oxide (rGO); (**c**) Multi-walled carbon nanotubes (MWCNT); (**d**) Carbon black (CB) particles.

**Figure 4:**
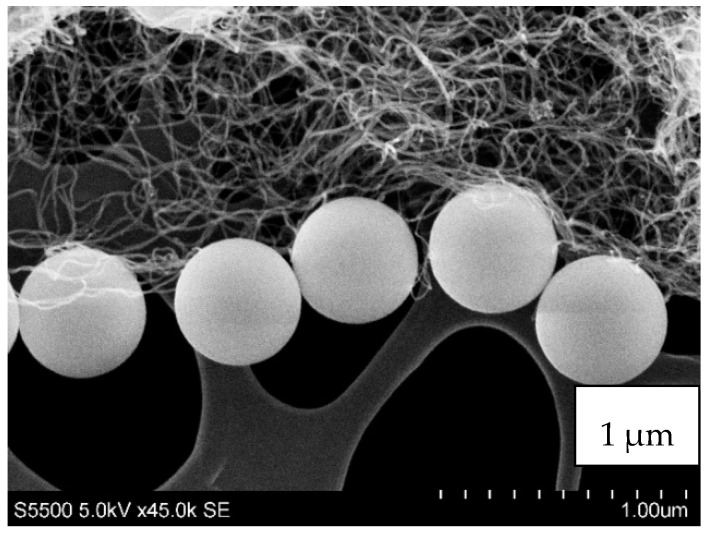
The shape and size of the MWCNT and spherical fumed silica KE-P50.

The carbon additives for the STFs and amorphous silica were subjected to SEM observations using a HITACHI S3500 scanning electron microscope (SEM) in SE mode (Figure 3 and Figure 4). It can be seen that all the particles used for testing have different shapes and various tendencies for agglomeration.

The particles of silica that were used for testing had a regular spherical shape with fairly uniform size distribution and low surface development (Figure 4). They had a slight tendency for agglomeration. The carbon fillers, in contrast, had a high tendency for agglomeration, forming bundles (especially MWCNT, Figure 3c). Graphene oxide and reduced graphene oxide exhibited flake shapes. Carbon black had an irregular shape also with the tendency for agglomeration.

**Figure 5:**
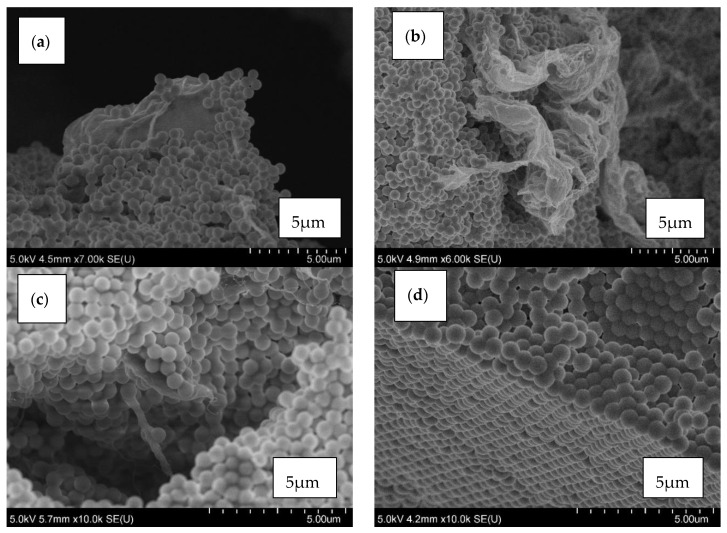
The arrangement of solid components in the shear thickening fluid (STF) with 55 vol.% of silica and 0.25 vol.% of (**a**) GO; (**b**) rGO; (**c**) MWCNT; (**d**) CB particles.

In Figure 5, the distribution of silica and carbon nanofillers in the STFs are shown. The fluids with the highest carbon fillers content, 0.25 vol.%, were degassed (approx. 0.08 MPa), at 100 °C to determine the distribution of the solid components.

Mixing of fumed silica with carbon nanofillers in a carrier liquid leads to breaking the agglomerates of the fillers and as a result they are more evenly distributed among the silica particles [23,24,28]. This effect strongly depends on the filler used. The GO and rGO behave similarly, i.e., they are distributed between the silica particles (Figure 5a,b). Numerous agglomerates are also visible.

In Figure 5c, the distribution of silica and MWCNT in the STF is shown. The single tubes can be seen. Mixing of fumed silica with MWCNT leads to breaking of the agglomerates of the nanotubes and their even dispersion in the voids between the silica particles. The agglomerates of CB were destroyed (Figure 5d) and they are not visible in the image. The crushed CB particles could be placed between the silica spheres.

### 3.2. Rheological Properties

**Figure 6:**
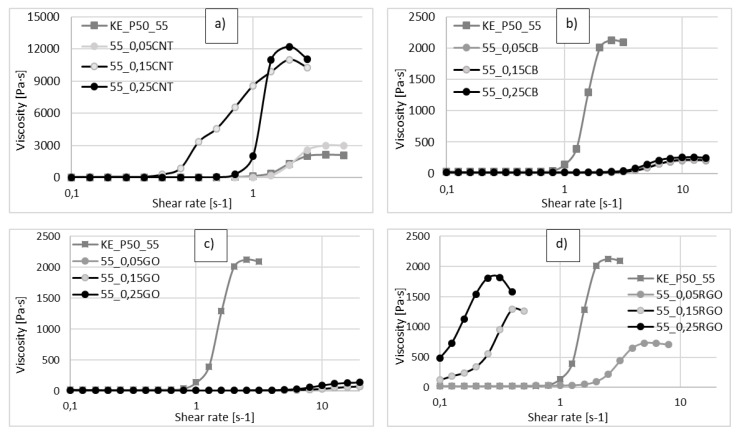
Viscosity versus shear rate for different volume fractions of carbon fillers. (**a**) CNT; (**b**) CB; (**c**) GO; (**d**) rGO), as compared with STF having 55 vol.% of KE-P50 without additives.

In Figure 6, the rheological properties of composite STFs with various additives are presented. When a drastic increase of the viscosity begins, the viscosity and the critical shear rate strongly depend on the type and content of the carbon nanofiller. Increasing the carbon fillers content moves the critical shear rate to lower values. It means that the shear thickening effect occurs more easily. The values of the zero shear viscosity (z-s-v), obtained for the suspensions, except for the STF with 0.25 vol.% of rGO, are similar (not higher than 70 Pa·s). With an increase of the shear rate, an abrupt increase of the viscosity is observed. The greatest effect of the dopant on the maximal viscosity is observed for the MWCNT. The highest values of the viscosity were obtained for 0.15 vol.%, 10,994 Pa·s, and for 0.25 vol.%, 12,213 Pa·s, with a critical shear rate below 2 s^−1^ for both fluids. The latter value is over five times higher than the result for the MWCNT-free fluid.

The lowest values of maximal viscosity were obtained for the samples with GO and CB (not higher than maximal viscosity for the pure STF, equal to 2127 Pa·s (Figure 6b,c). The viscosity values are low and the changes of the viscosity occur at higher shear rates, which can be explained by the fact that these additives react as a lubricant for the movement of the silica particles. The GO and CB cause the silica particles to move freely without being blocked. When the shear rate increases, the particles of CB and GO slightly retard the movement of silica particles, leading to a small thickening effect. However, this effect is not as strong as in the case of pure STF. Greater values were obtained for rGO (Figure 6d), however, the highest value of the maximal viscosity is still lower than the result achieved for the pure STF.

The rheological properties of the STFs depend on the ability of the silica particles to move against each other. This ability depends on the volume fraction of the solid components and their mechanical properties. The effect of a minor addition of carbon nanostructures is not fully clear. However, from the results presented, we can conclude that the addition of the carbon nanofillers increases the initial packaging of the solid components, and thus the (z-s-v) viscosity (GO, rGO). In the course of calendering and mixing, the structure of the carbon nanofillers becomes partially destroyed and located on the surface of silica particles and voids between them. Depending on its form, the carbon layer can act as either a lubricant or a friction increasing factor that retards the movement of the silica spheres. The latter behavior was observed for MWCNT. Apparently the MWCNT, due to their characteristic nanostructure and high mechanical properties, promote blocking of the silica particles movement. We assume that mixing the amorphous silica with MWCNT leads to breaking the agglomerates of the nanotubes. In the consequence of this process, the individual MWCNT can be dispersed in the voids between the silica particles, leading to increased friction between the particles and improved blocking of their movement.

### 3.3. Impact Absorption Ability

The STFs with 55 vol.% of amorphous silica and with the addition of carbon nanotubes were used for carrying out an absorption ability test. During the tests, none of the samples were destroyed.

**Figure 7:**
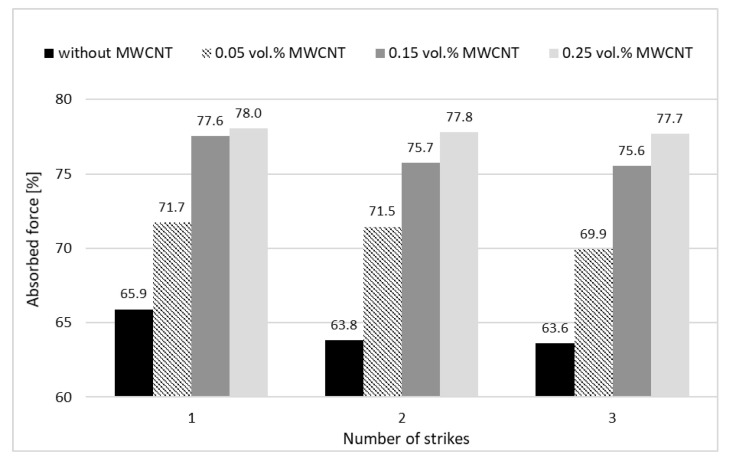
The percentage of absorbed force for STFs sealed in silicone forms after one, two, and three strikes at the same point, with various additions of MWCNT.

Figure 7 presents the values of the absorbed force for STFs sealed in silicone form after one, two, and three strikes at the same point with the addition of MWCNT. One can see that a higher addition of MWCNT provides an increase of the absorbed force. The samples containing 0.25 vol% MWCNT show the highest absorbing properties (78% for the first strike). The lowest value of absorbed force, for the first strike, was obtained for STF without nanofillers (approximately 66%).

The mean value of the stability of the absorbed force after three strikes is satisfactory for all the samples. The changes after all the strikes (comparison between first and third strikes) are not greater than 3.5% for pure STF and approximately 0.5% for the sample with 0.25 vol.% of MWCNT. It means that the composite STFs provide high protection ability and also stability of the properties after three strikes at the same point. This fact is related to the specific mechanism of the protection, which is due to the reversible rearrangement of the solid particles within the STF structure.

### 3.4. Structural Stability

**Figure 8:**
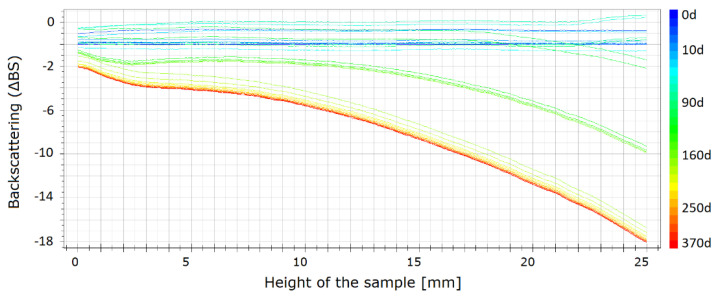
Backscattering for the STFs having 55 vol.% of KE-P50.

**Figure 9:**
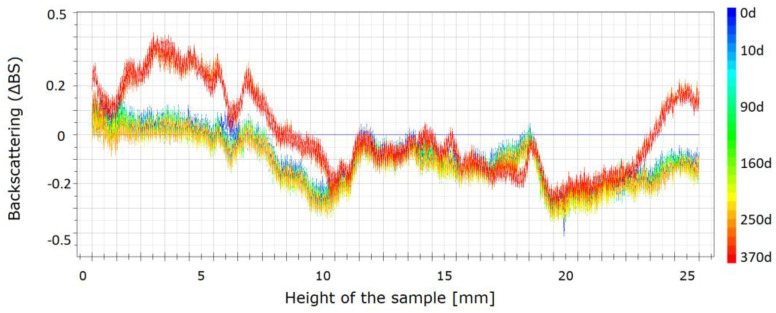
Backscattering for the STFs having 55 vol.% of KE-P50 and 0.25 vol.% of rGO within 370 days.

**Figure 10:**
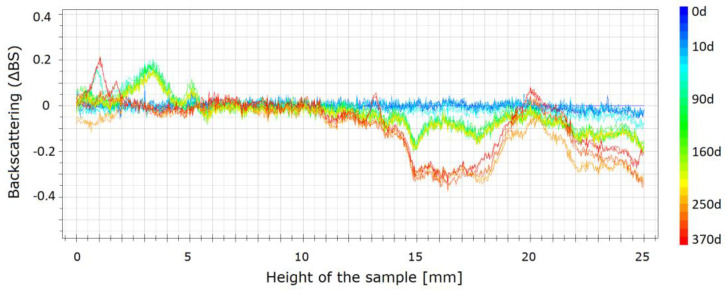
Backscattering for the STFs having 55 vol.% of KE-P50 and 0.25 vol.% of MWCNT within 370 days.

Usually, the published results for the stability studies of the STFs suspensions are based on optical observations of sedimentation in typical glass vials at room temperature [29]. However, such data are only comparative and do not allow for an accurate, quantitative comparison of the fluids. In the current study, the stability was measured using the Turbiscan LAB. The backscattered profiles were obtained for three fluids, i.e., one without carbon nanofillers and two with carbon additives. Because the values obtained for rGO, GO, and CB were similar, for the comparison, only the results for MWCNT and rGO are presented. The changes of the stability are presented in Figure 8, Figure 9 and Figure 10. The spectra reflect the backscattered light flow in relation to the sample height (mm). The time of the measurement is represented by colors of the lines. Different colors of lines present destabilization of the structure of the STF. The blue color shows the beginning of the measurements and the red color shows the end of the measurement.

As shown in Figure 8, changes in the pure STF are visible. It should be mentioned that, after one year, a slight destabilization occurs through the entire vial. The mean values of backscattering are approximately 20, which means that the particles change their position slightly and as a result the properties of the STF change slightly. The values obtained for both samples with the carbon additives are shown in Figure 9 and Figure 10. The absolute value of the change does not exceed one. It means that the changes are insignificant. For the sample with MWCNT, these changes are lower than for rGO. The changes are not higher than 0.4. It means that the addition of MWCNT provides the best structural stability of the fluids over time.

## 4. Conclusions

The composite STFs based on amorphous silica with polypropylene glycol and four types of carbon nanofillers were fabricated. Multi-walled carbon nanotubes, reduced graphene oxide, graphene oxide, and carbon black were applied as the carbon nanofillers. The microstructure, rheological properties and structural stability over time were studied and discussed. It was found that the MWCNT provide the most significant changes in the rheological properties of the STF, resulting in the highest maximal viscosity obtained in the rheological tests (almost five times greater than the result for the MWCNT-free fluid). We assume that, depending on the form of the carbon additive, it can act as either a lubricant or a friction increasing factor that retards the movement of the silica particles. Apparently the MWCNT, due to their characteristic nanostructure and high mechanical properties, increase the friction and promote blocking of the silica particles movement.

The carbon nanofillers lead to an increase of the structural stability of the STFs. The mean values of backscattering for pure STF are approximately 20 times higher than for STFs with carbon nanofillers, which means that STFs with carbon additives are more stable than the pure STFs.

The addition of the MWCNT also has a significant influence on the impact force absorbing efficiency. The silicone structure containing 55 and 0.25 vol.% of fumed silica and MWCNT, respectively, is able to absorb up to 78% of the impact force. Comparing with the structure with pure STF, this value is approximately 12% higher after the first strike.

The STFs containing MWCNT have great potential for application in smart protective structures, such as sport protective garments, liquid body armor, packaging systems and many other applications.

## Figures and Tables

**Table 1:** The properties of carbon nanofillers.

Fillers	Density (g/cm^3^)	Carbon Content (%)	Parameters (nm)	Specific Surface Area (m^2^/g)
MWCNT	2.08	>90	9.5 (diameter) 1500 (length of a single tube)	300
CB	2.05	>95	11 (particle size)	350
GO	2.03	40	0.9 (distance between surfaces)	211
rGO	2.37	75	0.37 (distance between surfaces)	266
